# A novel method for the quantification of key components of manual dexterity after stroke

**DOI:** 10.1186/s12984-015-0054-0

**Published:** 2015-08-02

**Authors:** Maxime Térémetz, Florence Colle, Sonia Hamdoun, Marc A. Maier, Påvel G. Lindberg

**Affiliations:** FR3636 CNRS, Université Paris Descartes, Sorbonne Paris Cité, 75006 Paris, France; Service de Médecine Physique et de Réadaptation, Université Paris Descartes, Hôpital Sainte-Anne, 75014 Paris, France; Centre de Psychiatrie et Neurosciences, Inserm U894, 75014 Paris, France; Université Paris Diderot, Sorbonne Paris Cité, 75013 Paris, France

**Keywords:** Stroke, Hand, Finger, Dexterity, Force, Finger tapping, Independence of finger movements

## Abstract

**Background:**

A high degree of manual dexterity is a central feature of the human upper limb. A rich interplay of sensory and motor components in the hand and fingers allows for independent control of fingers in terms of timing, kinematics and force. Stroke often leads to impaired hand function and decreased manual dexterity, limiting activities of daily living and impacting quality of life. Clinically, there is a lack of quantitative multi-dimensional measures of manual dexterity. We therefore developed the Finger Force Manipulandum (FFM), which allows quantification of key components of manual dexterity. The purpose of this study was (i) to test the feasibility of using the FFM to measure key components of manual dexterity in hemiparetic stroke patients, (ii) to compare differences in dexterity components between stroke patients and controls, and (iii) to describe individual profiles of dexterity components in stroke patients.

**Methods:**

10 stroke patients with mild-to-moderate hemiparesis and 10 healthy subjects were recruited. Clinical measures of hand function included the Action Research Arm Test and the Moberg Pick-Up Test. Four FFM tasks were used: (1) Finger Force Tracking to measure force control, (2) Sequential Finger Tapping to measure the ability to perform motor sequences, (3) Single Finger Tapping to measure timing effects, and (4) Multi-Finger Tapping to measure the ability to selectively move fingers in specified combinations (independence of finger movements).

**Results:**

Most stroke patients could perform the tracking task, as well as the single and multi-finger tapping tasks. However, only four patients performed the sequence task. Patients showed less accurate force control, reduced tapping rate, and reduced independence of finger movements compared to controls. Unwanted (erroneous) finger taps and overflow to non-tapping fingers were increased in patients. Dexterity components were not systematically related among each other, resulting in individually different profiles of deficient dexterity. Some of the FFM measures correlated with clinical scores.

**Conclusions:**

Quantifying some of the key components of manual dexterity with the FFM is feasible in moderately affected hemiparetic patients. The FFM can detect group differences and individual profiles of deficient dexterity. The FFM is a promising tool for the measurement of key components of manual dexterity after stroke and could allow improved targeting of motor rehabilitation.

## Background

Manual dexterity can be considered as the ability to perform accurate and coordinated hand and finger movements, such as fine control in grasping and manipulating small objects. Across species, manual dexterity is most evolved in humans [1]. This high degree of manual dexterity is made possible by specializations in hand morphology (skeletal, muscular) and neural control (corticospinal tract) [2]. Together these specializations allow for purposeful goal- and object-oriented manual control. There is, however, no consensus on how dexterity should be operationally defined and quantified. Although historically an ‘index of dexterity’ was developed (mainly for phylogenetic considerations [3]), it has become clear that behavioral dexterity cannot be defined by a single variable. Consequently, several studies have outlined key components of manual dexterity in terms of motor control: (i) Control of force, such as the capacity to control the force in each finger [4], in precision grip [5], in power grip [6, 7] and during grasp-and-lift tasks [8], (ii) Finger independence, i.e. the capacity to move the fingers independently of each other [9, 10]. (iii) Timing aspects, illustrated by the capacity to synchronize finger movements [11] and (iv) Motor sequence performance, i.e., activation of different fingers in a temporal sequence [12, 13]. However, a simultaneous description of such components is lacking in patients with neurological upper limb impairments.

Stroke is the first cause of acquired handicap in adults and about 50 % of stroke survivors have impaired upper limb and hand function in the chronic phase [14, 15], which impacts strongly on activities of daily living and on independence. Most of the above outlined dexterity components have been studied in stroke patients: (i) In terms of force control: post-stroke upper limb weakness is prevalent [14, 16, 17] and a decrease of accuracy in force control has also been reported (power grip [18]; precision grip: [19]; grasp-and-lift tasks: [20, 21]). (ii) Studies have also shown decreased independence of finger movements and increased motor overflow after stroke [22, 23]. (iii) Timing is also impaired after stroke: repetitive finger movements are slowed down and regularity is decreased [24–26]. (iv) Execution of sequential finger movements can also be compromised in stroke [27]. Therefore, manual dexterity can be impaired by decreased control of force, lower independence of finger movements, slowed timing or deficient finger sequencing.

In spite of evidence of impaired components of dexterity, clinical practice in terms of diagnosis and treatment of manual dexterity relies essentially on ‘functional’ measures and scales. Although largely applied, some of these scales are subjective, show questionable validity and reliability [28, 29] and some have high measurement error [30]. This may hamper detection and evaluation of motor deficits and affect evaluation of spontaneous or treatment-specific recovery [31, 32]. Most critically: usually these methods assess only one of the components of dexterity. It remains therefore unclear to what degree each of these components is affected in hemiparetic patients with impaired hand function.

In this study, we aimed at quantifying key components of manual dexterity and to describe how these components are affected after stroke. To this purpose we developed a new device (the Finger Force Manipulandum, FFM) designed to quantitatively assess several key components of dexterity. The objectives of the study were (i) to test the feasibility of using the FFM to assess manual dexterity components in stroke patients with impaired upper limb function, (ii) to investigate differences between stroke patients and healthy subjects, and (iii) to describe individual profiles of key components of manual dexterity in stroke patients.

## Methods

### Subjects

Ten adult stroke patients were recruited at the Rehabilitation clinic at Sainte-Anne Hospital, Paris. All patients suffered from a single ischemic or hemorrhagic stroke and were at least 2 weeks post-stroke at the time of their participation to the study. Included patients had varying degrees of hemiplegia affecting the upper limb and the hand. Exclusion criteria comprised severe loss of sensation of the affected limb, other neurological conditions and cognitive dysfunction that would interfere with the understanding of the experiment, such as visual deficits or severe neglect. Ten healthy control subjects, comparable in age, were also recruited. Table 1 lists the demographic and clinical details. The procedures of the study complied with the Declaration of Helsinki, and subjects provided informed consent.

**Table 1:** Clinical measures

Participant Patients	Age	Gender	Lesion location	Hemi-paretic side	Etio-logy	Time since lesion (days)	ARAT (max = 57)	Max. grip force (kg) affected\non-affected	% max. grip force (%)	Moberg Pick-Up Test (s) affected\non-affected	Mono-filaments (g) affected\non-affected
1	76	F	Right precentral gyrus and right lenticular nucleus	left	H	36	57	12\16	75	16\12	0.4\0.4
2	49	M	Left parieto-occipital cortex, intra-ventricular and corpus callosum	right	H	120	57	42\39	100	25\13	0.4\0.07
3	25	M	Right temporo-parietal cortex	left	H	330	32	15/44	34	60\13	0.07\0.07
4	68	F	Left fronto-parietal cortex	right	I	19	57	11/15	73	19\14	0.07\0.4
5	46	M	Right sylvian and subdural hematoma	left	I	165	51	12/26	46	50\30	0.4\0.4
6	68	M	Left sylvian	right	I	315	40	18/37	49	60\22	0.07\0.4
7	40	M	Left thalamus	right	H	75	40	6/43	14	51\21	0.07\0.4
8	64	M	Left pons	right	I	40	57	38/30	100	32\24	0.4\0.4
9	50	F	Left precentral cortex and left semi-oval center	right	I	210	56	19/24	79	13\12	0.4\0.4
10	65	M	Left pons	right	I	180	57	17/39	43	26\17	0.4\0.4
Patients	55.1 (±15.7)	3 F/7M				149 (±112)	50.4 (±9.4)	31.3 (±10.7)	61.3 (±28.5)	35.2 (±18.3)	0.33 (±0.14)
Mean (±SD)								\19.0 (±11.7)		\17.8 (±6.2)	\0.27 (±0.18)
Controls Mean (±SD)	52.9 (±17.4)	4 F/6 M						35.1 (±11.4)		14.3 (±1.9)	0.14 (±0.14)

For each stroke patient is indicated: age, gender, lesion location, hemiparetic side, etiology (type of stroke: H = hemorrhagic; I = ischemic), time since lesion (days), total ARAT (Action Research Arm Test) score, MVC grip force in kg in the hemiparetic and non-affected hand, % MVC in the hemiparetic hand compared to the non-paretic hand, performance of the Moberg pick-up Test (time in s) for both hands, % of proprioception, and tactile sensibility (Semmes-Weinstein mono-filament test) for both hands. Bottom two lines: mean and standard deviation in stroke patients and control subjects

### Clinical measures

The Arm Research Action Test (ARAT), a clinical test for grasp, grip, pinch and gross movement in the hemiparetic hand, was used as a global measure of hand function [33, 34]. The Moberg pick-up test was used as a clinical assessment of grip function in each hand. Time taken to place all 12 objects into the box was recorded. The time taken reflects the degree of precision grip function (>18 s is considered pathological in this age span) [35]. A Semmes-Weinstein mono-filament test with three calibers (2 g, 0.4 g and 0.07 g) was used to measure the tactile sensitivity of finger tips in each hand [36]. Maximal grip force (in Kg) in each hand was recorded (best of two trials) with a hydraulic Jamar dynamometer (http://www.lafayetteevaluation.com). Proprioception was tested by assessing the subjects’ capacity to detect and match passive finger displacement in one hand while keeping the eyes shut and rated as intact, impaired or absent. All measures were also obtained in control subjects, except the ARAT.

### Finger Force Manipulandum (FFM)

**Fig. 1:**
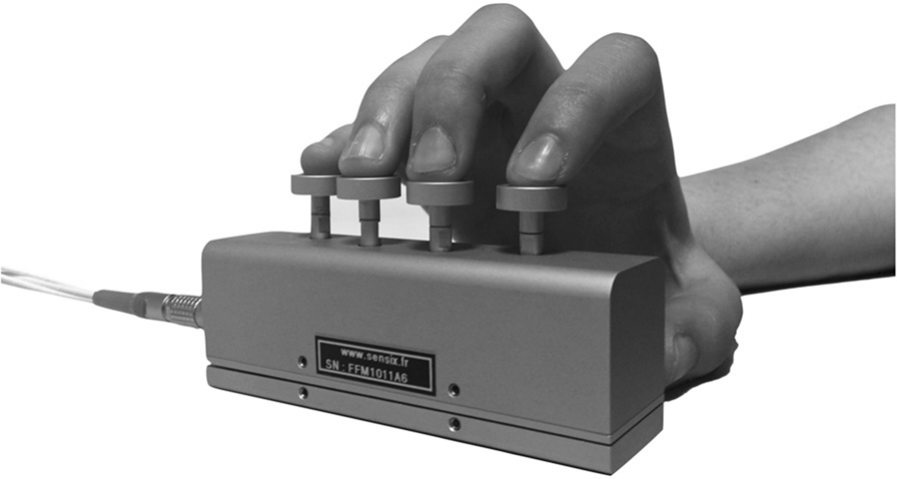
The Finger Force Manipulandum (FFM). Index, middle, ring and little finger each apply forces on a spring-loaded piston. Two types of tasks were implemented: continuous force tracking and finger tapping. Forces applied by each finger were recorded via a CED interface (not shown) and used for real-time visual feedback and for performance analysis

Together with Sensix (www.sensix.fr) we developed the Finger Force Manipulandum (FFM) in order to quantify key components of manual dexterity in stroke (and other) patients. The FFM is equipped with four pistons positioned under the tip of the index, middle, ring and little finger, each coupled to an individual strain gauge force sensor (Fig. 1). The height of the pistons can be adjusted but in this study we used a constant piston height of 15 mm across all subjects. Pistons have a contact surface of 15 mm diameter and are 20 mm apart. With increasing force the pistons move against a spring load over a range of 10 mm. The end of this dynamic (non- static) range is reached with 1N. Above 1N, forces are controlled isometrically. Thus each sensor measures force along the piston axis exerted from each finger independently. The precision of the sensor is <0.01N, with a range of 0–9N. Force data of each finger was sampled to a CED 1401 (with 10 kHz sampling rate/digit) connected to a computer running Spike 2v6 (Cambridge Electronic Design, www.ced.co.uk) software. Custom-written CED scripts provided real-time visual display of digit forces and target instructions or target forces.

### FFM tasks

Four separate tasks (i-iv) were developed in order to quantify different components of manual dexterity. The finger force tracking task was developed in order to measure the capacity to generate and control fingertip forces [18]. The sequential finger tapping task was developed in order to assess the ability to learn and recall finger movement sequences [37]. The single finger tapping task is a timing task designed to test the capacity to perform repetitive tapping with and without auditory cues [9]. The multi-finger tapping task was designed to test the independence of finger movements in one-finger configurations [22, 38] and two-finger configurations. Each of the four tasks comprised different conditions in order to evaluate performance across varying forces, tapping frequencies, and fingers. In all tasks the subject was first required to place the fingers on the pistons and was instructed to maintain the fingers on the pistons throughout the tasks. Every subject was able to use the FFM with the forearm supported on the table and the shoulder was in a relaxed slightly flexed position. To ensure a comfortable position some subjects used a silicone wrist support during the tasks.

**Fig. 2:**
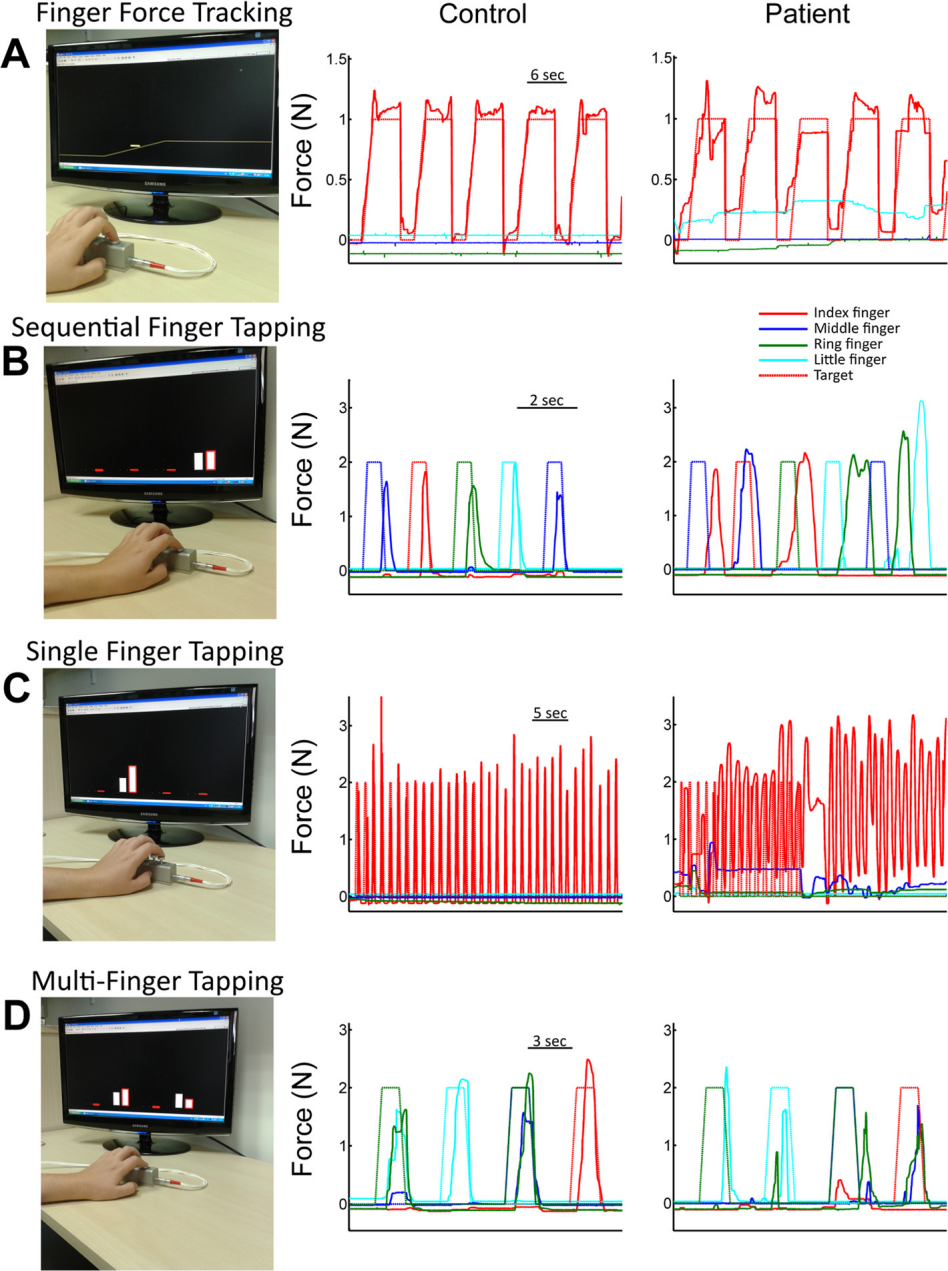
The four FFM tasks. **a**-**d**: Left panels: Setup with FFM and screen providing visuo-motor feedback. Right panels: Example recordings of finger force traces. Index finger: red, middle: blue, ring: green, little: turquoise. The target for each finger is shown as a line of the same color (trapezoid form in **a**, **b**, **d**). Left column: control subject. Right column: stroke patient. **a** *Finger force tracking*. Screen: The yellow line represents the target force and the cursor (here close to the ramp) represents the instantaneous force exerted by the index finger. The subject has to match the vertical cursor position with the target force. Right panels: tracking examples of five successive trials. Note: the patient’s tracking force trace is more irregular, does not return to baseline between trials and the little finger (turquoise) applies unwanted force (motor overflow). **b** *Sequential finger tapping*: Screen: the height of 4 red vertical bars represents the force exerted by each finger. Next to each finger feedback the target bar (white), here only visible for the index finger. Successively appearing target bars indicate the 5-tap finger sequence (e.g., digit 3-2-4-5-3). Right panels: correct tapping sequence for the control subject, erroneous sequence in the patient. **c** *Single finger tapping*: Screen: ring finger is indicated as tapping finger (white bar). Visual feedback was only provided for the tapping finger (red bar). Right: index finger 1Hz condition with (15 s) and without (20s) tapping cue. Less finger taps, incomplete return to baseline and unwanted movements of other fingers are noticeable in the patient. **d**) *Multi*-*finger tapping*: Screen: two-finger target tap (index and ring finger, white bars) and corresponding two-finger user tap (red bars). Right: four subsequent trials, each with a different finger combination (ring-little; little; middle-ring; index). The patient clearly has more difficulties

(i) The *Finger Force*-*Tracking* task is a visuo-motor task of finger force control. By varying the force on the piston with the finger, the subject controlled a cursor on a computer screen (Fig. 2a). The subject was instructed to follow the target force as closely as possible with the cursor. The target force (a line) passed from right to left over the screen, presenting successive trials. Each trial consisted of a ramp phase (a linear increase of force over a 1.5 s period), a hold phase (a stable force for 4 s) and a release phase (an instantaneous return to the resting force level, 0N) followed by a resting phase (2 s). Trials were repeated 24 times, distributed in four blocks of 6 trials, two blocks with a target force of 1N and two with a target force of 2N. These low absolute forces were chosen since dexterous action usually employs low forces at which key sensory events occur [39]. In this study, patients performed the finger force-tracking task separately with the index and the middle finger of their hemiparetic hand and controls performed the task with their index and middle finger of their right hand. Task duration was 3 min 20 s/digit.

(ii) The *Sequential finger tapping* task is a 5-tap finger sequence involving the four digits. The visual display consisted of 4 columns (representing the 4 digits), whose height varied in real-time as a function of exerted finger force (feedback). In addition, a target column (cue) adjacent to each feedback column indicated the piston to be pressed (Fig. 2b). The subject was instructed to press the indicated piston as soon as the target appeared. The 5 successive targets of a given sequence appeared at a rate of 1 Hz. Each sequence was repeated 10 times with visual cues (learning phase) and then repeated 5 times from memory, i.e. without cues, and as quickly as possible (recall phase). Force feedback was always present. Subjects were instructed to match the tap force approximately to target of 2N (same for the other tapping tasks). In this protocol, the subjects performed three previously unknown motor sequences: they first learned and then repeated the sequence (A) 2-5-3-4-2 (2 = index; 5 = little); then the sequence (B) 4-3-5-2-4 and finally the sequence (C) 3-2-4-5-3. A single sequence (trial) of 5 taps lasted 5 s and the duration for all 15 trials was 2 min 20 s.

(iii) The *Single finger tapping* task consisted of repetitive tapping with one finger with or without an auditory cue. The visual display was similar to that in task (ii) and indicated which finger to tap but did not provide any timing cue. Three tapping rates were tested: 1, 2 and 3Hz (similar to [9]). After the cued tapping period (15 taps) the subject was required to continue tapping for a similar period, without cue but at the same rate. The subject started at 1 Hz with the index finger, followed by the middle (Fig. 2c), ring and little finger. This protocol was repeated at 2 Hz and then at 3 Hz. The total duration of this task was 4 min.

(iv) The *Multi*-*finger tapping* task consisted of simultaneous tapping with different finger configurations in response to visual instructions. The visual display was similar to that in task (ii) and (iii). Subjects were instructed to reproduce 11 different finger tap configurations following the visual cue (Fig. 2d). The 11 different configurations consisted of 4 one-finger taps (separate tap of index, middle, ring or little finger), 6 two-finger configurations (simultaneous index-middle, index-ring, index-little, middle-ring, middle-little or ring-little finger taps), and one four-finger tap. All configurations were performed twice resulting in a total of 32 (4 × 8) one-finger taps, 30 (6 × 5) two-finger taps and 2 four-finger taps. Performance measures were calculated for one and two-finger configurations. Four finger taps were not analyzed. The order of the configurations was pseudo-randomized with equal number of transitions between one and two-finger taps. The entire task with its 64 trials lasted 4 min and 40 s.

### Data analysis

Task performance was analyzed using Matlab (v7.5, The MathWorks, Inc., Natick, MA, USA). The four force signals were first down-sampled to 100 Hz for the analysis.

*Finger force tracking*: all performance measures were calculated trial-by-trial (N = 24). Tracking error was calculated as the root-mean-square error (RMSE) between the actual applied force and the target force. The error was separately extracted during the ramp and the hold phase. The time of the force onset in response to the target ramp and the time of the release onset at the end of the hold phase were calculated as threshold crossings of dF/dt. The release duration was computed as the time taken to reduce the force from 75 to 25 % of the target force [18]. The coefficient of variation (CV) of force (i.e. SD/mean across time bins) was calculated during the hold phase and averaged across trials. Mean force during the hold was calculated as the average force across 3 s excluding the first and last 500 ms of the hold phase. Mean baseline force was calculated as the average force during the resting phase between each trial from 1500 ms to 500 ms before the ramp onset.

For the *three tapping* tasks the finger taps were identified in a similar way. Starting from the force trace each tap was identified as a discrete event according to threshold (>0.5N) allowing identification of target and the applied force peaks (retained as taps). The time location and amplitude of each tap were then recorded. Subsequently, the following task-specific performance variables were obtained:

In the *Sequential finger tapping* task we computed the number of user taps trial-by-trial, i.e. for each 5-tap target sequence. By comparing the user taps to the target sequence, each trial was then labeled as correct or incorrect. In case of an incorrect sequence the number of missing or additional unwanted taps was recorded, as well as the number of consecutive correct taps within parts of the sequence. Furthermore, performance was calculated across trials, by computing the number of correct trials and the number of error taps for each finger. These measures were obtained for the learning and the recall phase, respectively.

In the *single finger tapping* task the lead-finger (target finger) and the non-lead-fingers were identified in each condition (finger and 1, 2 or 3 Hz). For the lead-finger the number of taps, the tap amplitude, and the interval between consecutive taps were calculated for each condition. Unwanted taps were identified in the non-lead-fingers and labeled as overflow taps (non-lead-finger tap at the same time as a lead-finger tap) or as unwanted finger taps (non-lead-finger tap in the absence of a lead-finger tap). To estimate the capability to adapt the tapping rate to the target frequency of the cue we calculated the slope of the tapping rate across the 1 Hz, 2 Hz and 3 Hz conditions. A slope = 1 indicates correct tapping rate, a slope < 1 slower execution.

In the *multi*-*finger tapping* task each tap, in response to a displayed finger configuration, was identified as correct or incorrect (success rate), i.e. identical to or different from the required target taps. Errors, in each finger, were categorized as missing taps (omissions, omission rate), or as unwanted extra-finger-taps (one or several) (similar to errors reported in keyboard typing [40]). Across trials the number of errors was evaluated as a function of the target (one- or two-) finger configuration.

Finally, in order to obtain individual profiles of components of manual dexterity, we plotted each patient's performance in the six most discriminatory variables (showing group differences) and compared it to the performance range observed in the control group. Values beyond the control group's mean + 2SD in a given measure were considered indicative of pathological performance.

### Statistical analysis

Descriptive statistics are shown as mean ± SD. Student’s *T*-test was used to test for group differences in single-level variables. Differences in the measures obtained from the four tasks described above were tested using repeated measures ANOVAs. (i) Force tracking: independent variables (error, timing, etc.) were studied with ANOVA including one between-group factor GROUP (patients, controls), and three within-subject levels: FINGER (index, middle), FORCE (1N, 2N), PHASE (Ramp, Hold). (ii) Sequential finger tapping: independent variables (success rate, number of correct taps) were studied with ANOVA including one between-group factor GROUP (patients, controls), and two within-subject levels: SEQUENCE (sequence A, B, C), PHASE (learning and recall phase). (iii) Single finger tapping: independent variables (tapping rate, number of overflow taps, etc.) were studied with ANOVA including one between-group factor GROUP (patients, controls), and three within-subject levels: FREQUENCY (1, 2, 3 Hz), FINGER (index, middle, ring, little) and PHASE (with auditory cue, without auditory cue). (iv) Multi-finger tapping: independent variables (success rate, number of unwanted extra finger taps, etc.) were studied with ANOVA including one between-group factor GROUP (patients, controls). Post-hoc tests were performed using Fisher LSD Test. Spearman’s rank order correlation was used to investigate correlations between performance measures and clinical scores. Jamar and Moberg Pick up scores were presented as % of non-hemiparetic hand scores for correlation tests. Pearson’s correlation was used to test for relations between different performance measures. The level of significance was set to p < 0.05.

## Results

### Clinical assessment of hand and finger function

In stroke patients maximal power grip force in the paretic hand was significantly reduced to a mean of 19 kg compared to 35 kg in controls (P = 0.005). According to the ARAT, none of the patients were severely impaired (score < 5), five patients had moderately impaired hand function (51 < score < 57), and five scored the maximal 57 points [41]. However, three of these latter patients had reduced maximal grip force and four were slower in the pick-up test with the affected hand (Table 1). Sensory thresholds in the fingers were also significantly decreased in stroke patients (Table 1; P = 2 × 10^−10^) but only patient 3 had impaired proprioception.

### Task feasibility

All ten patients were able to accomplish the force tracking task and the single finger tapping tasks, and nine patients completed the multi finger tapping task. However, only four patients achieved the sequential tapping task since the rate of the target cue presentation (1 Hz) during the learning phase was too high. The main issues affecting feasibility were: maintaining all four fingers on the pistons and the sequential tapping task being too fast (Table 2).

**Table 2:** FFM ergonomic and task feasibility in hemiparetic patients

Patients	Ergonomic difficulties with the FFM device			Task feasibility				Problem encountered
	Arm posture	Finger position	Interaction with computer	Finger force tracking	Sequential finger tapping	Single finger tapping	Multi-finger tapping	
1	no	Maintaining little finger on piston (short little finger)	Difficulties to interact with the computer feedback	yes	no	yes	no	Too fast and difficult (sequence)
								Failed to use computer feedback (sequence and tapping)
2	no	no	no	yes	yes	yes	yes	/
3	Maintaining wrist extension (flexor spasticity)	Fingers slide on pistons (flexor spasticity)	no	yes	no	yes	yes	Too fast and difficult (sequence)
4	no	Maintaining little finger on piston (short little finger)	no	yes	no	yes	yes	Too fast and difficult (sequence)
5	no	no	Difficulties to interact with the computer feedback	yes	no	yes	yes	Too fast and difficult (sequence)
								Failed to use computer feedback (sequence)
6	Maintaining wrist extension (weak extensor)	Maintaining fingers on pistons (adductor spasticity)	no	yes	no	yes	yes	Too fast and difficult (sequence)
7	no	Maintaining little finger on piston (contracture of little finger)	no	yes	no	yes	yes	Too fast and difficult (sequence)
8	no	Maintaining fingers on pistons (repositioning)	no	yes	yes	yes	yes	/
9	no	no	no	yes	yes	yes	yes	/
10	no	no	no	yes	yes	yes	yes	/
Feasibility	8/10	4/10	8/10	10/10	4/10	10/10	9/10	/

Indicated are for each patient: qualitative observations in terms of ergonomic feasibility and task feasibility

### Force tracking

**Fig. 3:**
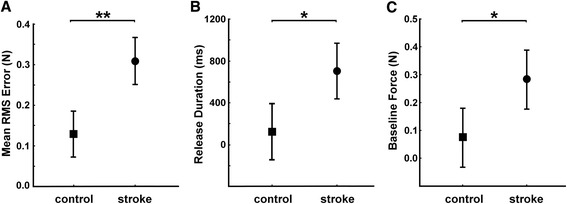
Finger force tracking. Group comparison between control subjects (square) and stroke patients (circle). **a**) Mean RMSE for index finger tracking (±95 % confidence interval) for ramp and hold phase combined. **b**) Mean release duration for trials at 1N and 2N with the index finger. **c**) Mean baseline force between trials. Asterisks indicate (here and in the following Figures) significant differences between the two groups, with * p < 0.05 and ** p < 0.01

Patients and controls applied the same amount of force during the hold phase in 1N (controls: 0.98N ± 0.2; patients: 1.1N ± 0.2; P = 0.24) and 2N conditions (controls: 1.9N ± 0.4; patients: 2.0N ± 0.2; P = 0.36). This task revealed dramatic differences in the precision of force control: stroke patients showed increased tracking error (0.31N ± 0.1) compared to controls (0.13N ± 0.06). This difference was highly significant (GROUP effect: F = 21.18; P = 0.0002; Fig. 3a) and was apparent during both the ramp and hold phases, and at both force levels (P = 0.01). Performance was equally impaired when using the index or the middle finger. Furthermore, time taken to release force at the end of the hold period (Fig. 3b) was significantly prolonged (about six times longer) in stroke patients (702 ms ± 557) compared to controls (123 ms ± 84) (GROUP effect: F = 5.03; P = 0.014). Patients also showed difficulty in not applying force (relaxing) with the lead-finger during the baseline (i.e. between trials, see Fig. 2a). The mean baseline force (Fig. 3c) was significantly different and about four times higher in patients (0.28N ± 0.21) compared to controls (0.07N ± 0.09; GROUP effect: F = 4.10; P = 0.028).

Some measures did not reveal any significant difference between groups: this was the case for the timing of the force onset (prior to the ramp) and for the release onset (at the end of the hold phase). Also the CV of tracking force was similar in the two groups.

### Sequential finger tapping

**Fig. 4:**
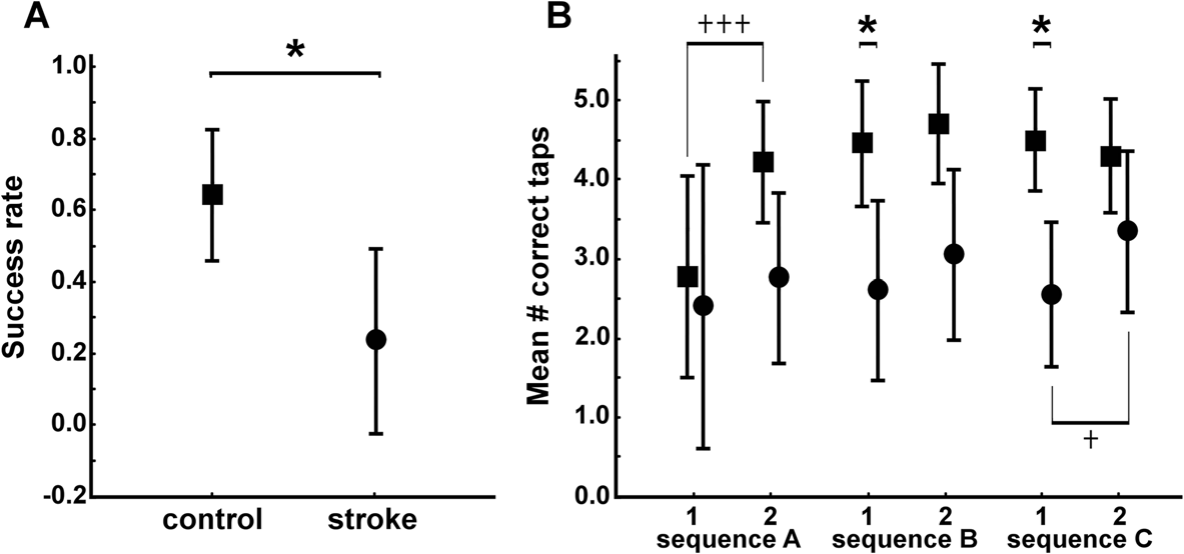
Sequential finger tapping. Group comparison between control subjects (square) and stroke patients (circle). **a** Mean success rate across all trials (learning and recall, sequence A, B and C) of the sequential finger tapping task. A success rate of 1 indicates perfect performance. **b** Mean number of correct taps (max = 5) for the first half (‘1’) and the second half (‘2’) of the learning phase of for each sequence (A, B and C). Note: patients and controls had similar numbers of correct taps at the first half of sequence A, controls subsequently increased their performance significantly (+++). In controls, learning during sequence A improved initial performance in subsequent sequences B and C: they had significantly more correct taps at the first halves of the sequences B and C (B: P = 0.04; C: P = 0.03) compared to patients. Significant differences between and within groups are indicated

The sequential finger tapping task turned out to be difficult for some patients. Control subjects achieved an average success rate of 0.66 ± 0.2, measured across all trials of the two conditions (learning and recall phases) and across the three different sequences (A, B, C). The four patients that accomplished this task reached a significantly lower success rate of 0.23 ± 0.28 (Fig. 4a, GROUP effect: F = 8.21; P = 0.017). Both groups showed similar performance in the first half of sequence A (Fig. 4b). During the learning phase (i.e. the cued condition), controls improved their performance by passing from a mean number of 2.7 (/5) correct taps to 4.2 (/5) between the first half and the second half of the learning phase for sequence A (P = 4 × 10^−6^; Fig. 4b). Controls showed maintained performance without obvious learning for the subsequent sequences B and C. In the patients significant improvement of performance between the first and the second half of the learning phase was only seen during the last sequence (sequence C): they passed from 2.5 (/5) correct taps to 3.4 (/5) (P = 0.02; Fig. 4b). In patients, no improvement was apparent during the first two sequences A and B. No significant group differences were found in the second halves of each sequence (Fig. 4b) nor in the recall phases.

### Single finger tapping

**Fig. 5:**
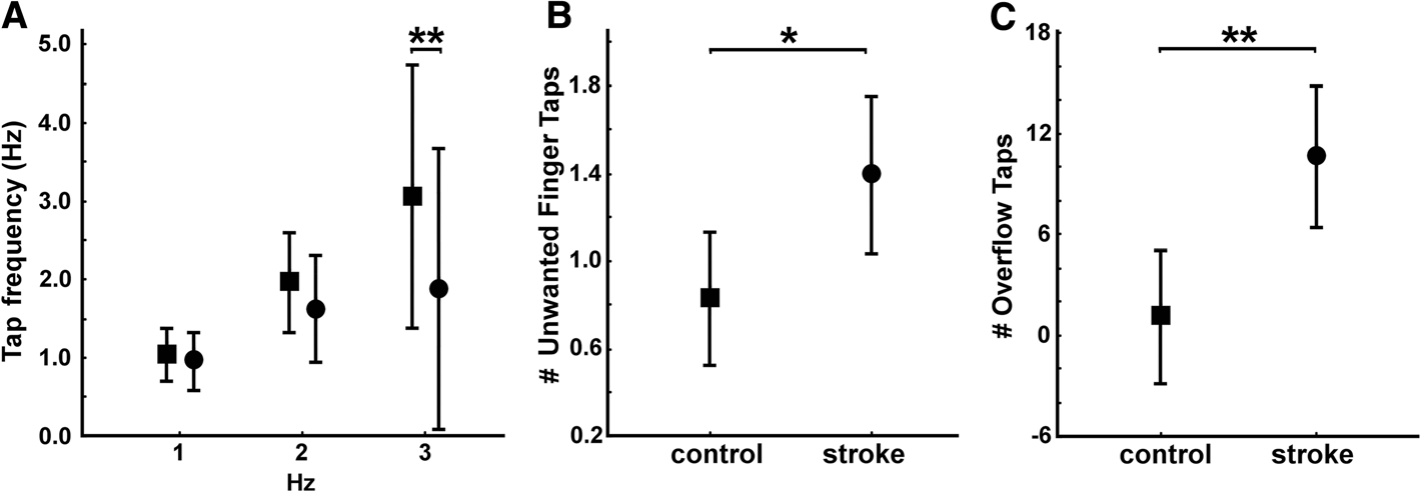
Single finger tapping. Group comparison between control subjects (square) and stroke patients (circle). **a** Mean tapping rate across all tested digits at 1 Hz, 2 Hz and 3 Hz. **b** Mean number of unwanted extra-finger-taps during each condition. **c** Mean number of non-wanted overflow taps across all conditions

We measured the average single finger tapping rate, cumulated over the cued and the non-cued condition (Fig. 5a). Controls were able to follow the imposed tapping rate, with a mean rate of 1.06 Hz ± 0.06, 1.98 Hz ± 0.13 and 3.17 Hz ± 0.47 for the 1, 2 and 3 Hz condition, respectively. The tapping rate was impaired in patients, with a reduced tapping rate of 2.31Hz ± 0.69 at the 3 Hz condition compared to controls (GROUPxFREQUENCY effect: F = 9.30; P < 0.001; post-hoc GROUP effect at 3 Hz: P < 0.001; but not at 1 or 2 Hz). Thus, patients had a decreased slope of tapping rate (1-3Hz) in all four fingers, with a grand average across fingers of 0.53 ± 0.36 compared to controls (1.05 ± 0.24; T = −11.2; P = 2 × 10 ^-9^). There was no difference in tapping rate between the cued and non-cued condition and no difference between fingers. No significant difference between groups was found in the tapping regularity, i.e., for the mean tap interval.

Unwanted finger taps occurred rarely during single finger tapping, i.e. a tap of a non-lead finger in the absence of a lead-finger tap. Per condition (Frequency/Finger: 35 taps) this occurred on average 0.8 times (0.8 taps/35) in controls, but significantly more often (1.4 taps/35) in patients (Fig. 5b, GROUP effect: F = 6.60; P = 0.021).

In patients the single finger tapping task also produced substantial unwanted motor overflow to fingers not involved in the task (i.e., non-lead finger taps concomitant with lead-finger taps). Patients showed significantly more overflow taps than controls (Fig. 5c, GROUP effect: F = 12.16; P = 0.003). At 1Hz patients made on average 10 extra overflow taps per condition (frequency/finger: for a total of 35 required taps) compared to a single overflow tap in controls. In both groups overflow taps were least frequent when the index or little finger acted as lead finger.

### Multi-finger tapping task

**Fig. 6:**
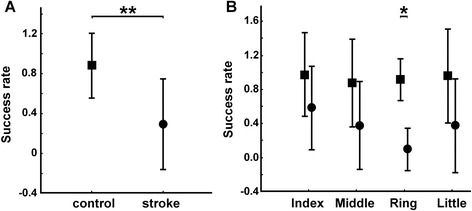
Multi-finger tapping. Group comparison between control subjects (square) and stroke patients (circle). **a** Mean success rate for each finger during one- and two-finger taps. **b** Mean success rate for each combination of finger(s) to activate (one or two fingers)

We first computed the average success rate across single- and two-finger combinations. Patients with a mean success rate of 0.3 ± 0.2 were less accurate compared to control subjects with a mean success rate of 0.9 ± 0.1 (Fig. 6a, GROUP effect: P = 4 × 10^−10^). This group difference was present in both one- and two-finger combinations (P = 3 × 10^−7^ and P = 1 × 10^−7^, respectively).

For one-finger taps, a FINGER × GROUP interaction was found (Fig. 6b, FINGER × GROUP effect: F = 5.90; P = 0.002). Posthoc testing showed significantly lower success rate in all four fingers in patients compared to controls with the ring finger most affected (with a success rate close to 0.1 for patients compared to 0.9 for controls; P = 2 × 10^−9^). For each failed one- or two-finger trial, we computed two types of errors: the omission rate and the number of unwanted extra-finger-taps. The omission rate was significantly greater in patients (0.2 ± 0.17) compared to controls (0.01 ± 0.01; GROUP effect: F = 12.24; P = 0.003). For one-finger conditions, a FINGER × GROUP interaction was found (FINGER × GROUP effect: F = 3.38; P = 0.03). Posthoc testing showed significantly higher omission rate in the ring and the little fingers in patients (with an omission rate close to 0.2 and 0.4 for patients compared to 0.01 for controls; P = 0.03 and P = 3 × 10^−5^). Summed across trials and fingers, unwanted extra-finger-taps were more frequent in patients (54 ± 24.1) than in controls (7.9 ± 6.9; T = 5.52; P = 0.0003).

**Fig. 7:**
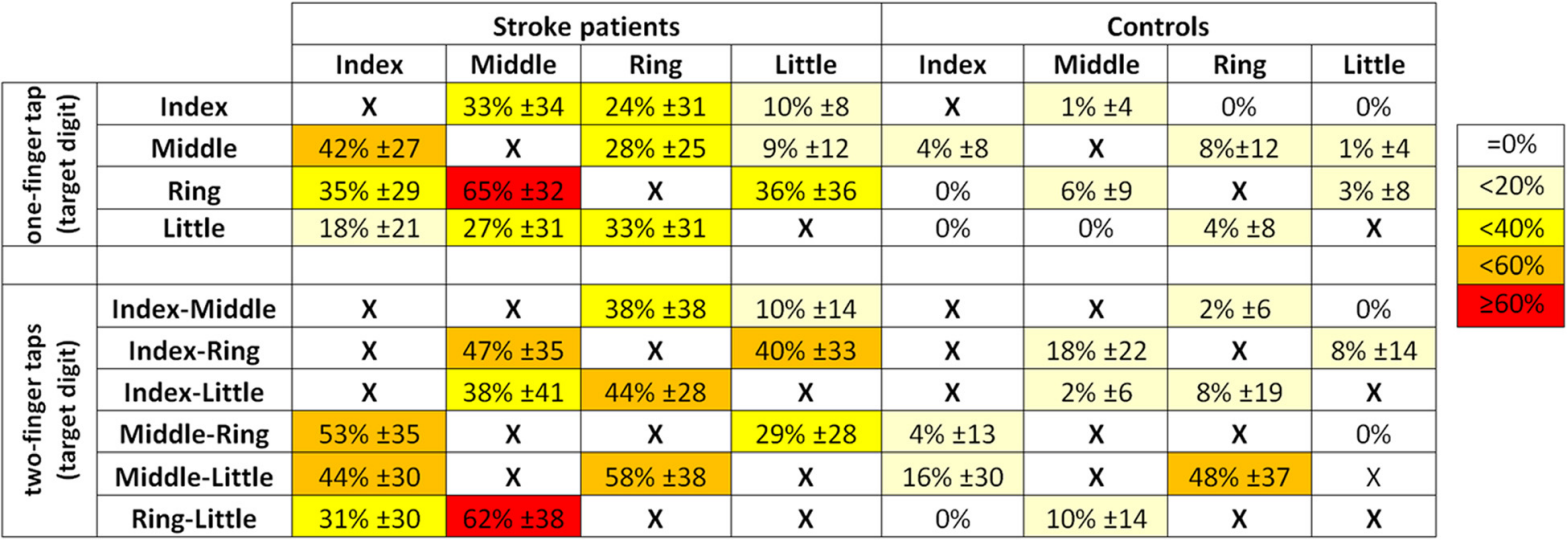
Finger tap errors as a function of target tap combination. Each line shows the occurrence of error taps during multi finger tapping. Error occurrence is given for each finger in % (mean ± SD) of target taps in the relevant condition for patients (left) and in control subjects (right). Example: in 10 % of all one-finger target taps with the index finger (target digit 2), patients also tapped erroneously with the little finger (digit 5). The first four lines describe each one-finger target tap condition, the following six lines every two-finger target tap combination. “Xs” indicate coincidence of target finger(s) and correct tap finger(s). Color scale indicates the level of error: white = no error (0 %), red > 60 % errors

The distribution of unwanted extra-finger-taps across fingers is shown in Fig. 7 for both one- and two-finger combinations. Each line in the Table shows the occurrence of unwanted extra-finger-taps as a function of finger combination. For every target combination, patients produced more error in other fingers than control subjects. In the least successful one-finger combination (the ring finger target tap) patients erroneously activated also the middle finger in more than sixty percent of the trials, while this was the case in less than ten percent in controls (Fig. 7). Note that the index and little finger also made errors in this condition, but less frequently (in about 35 %) than the middle finger. This same error pattern across fingers (i.e. middle finger error > index or little finger error) was also present in control subjects, but in an attenuated form. More generally, the pattern of unwanted extra-finger-taps formed a ‘neighborhood’ gradient, such that digits anatomically far from the target (lead) digit produced less error taps than those closer to (or immediate neighbors of) the target digit. This also held for the ‘2–3’ and ‘4–5’ two-finger combinations. Two-finger combination taps of non-adjacent digits (‘2–4’, ‘2–5’, ‘3–5’), showed, in absence of a distance gradient, a balanced error distribution. Similar but attenuated ‘across’ finger error patterns were also observed for the control subjects.

### Individual dexterity profiles

**Fig. 8:**
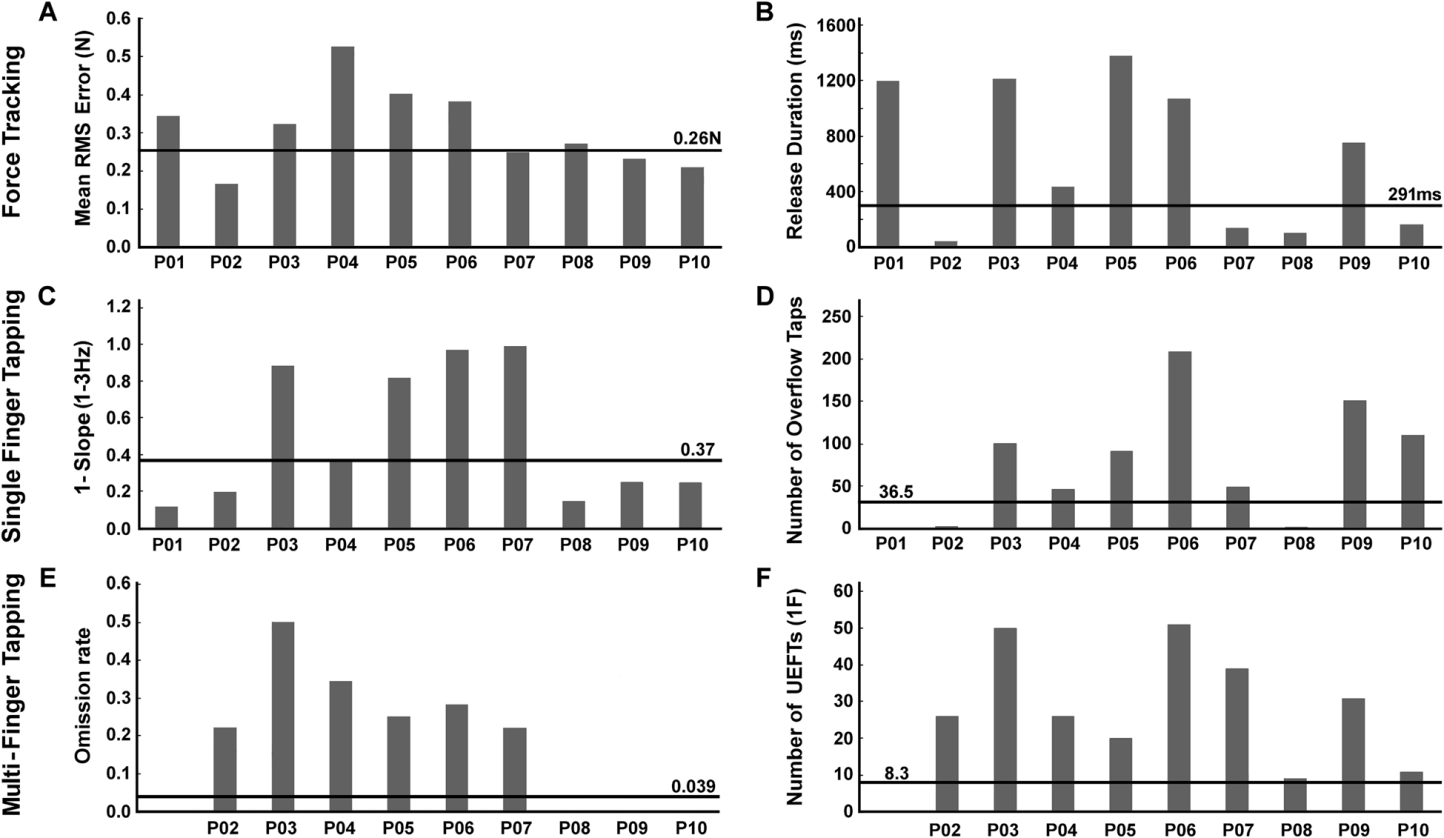
Individual dexterity profiles. **a**-**b** Force tracking, C-D) Single finger tapping, **e**-**f**) Multi-finger tapping. **a** Index finger force tracking: mean error score for each stroke patient (P01-P10). The ‘normality threshold’ (control average + 2SD) is indicated by a horizontal line (and its corresponding value). Individual scores > threshold were considered pathological. **b** Index finger force tracking: mean release duration. **c** Single finger tapping rate: 1 minus the slope 1-3Hz value for the index finger for each patient. **d** Single finger tapping: number of overflow taps during the 1Hz condition. **e** Multi-finger tapping: omission rate across all trials. **f** Multi-finger tapping: number of unwanted extra-finger-taps (UEFTs) for one-finger combination trials. Patient P01 did not perform this task

Individual profiles were investigated in six measures found to differ significantly between groups. From the tracking task we studied error and release duration. From the single-finger tapping task, slope of tapping rate and number of overflow taps were retained. And from the multi-finger tapping task, omission rate and frequency of unwanted extra-finger-taps were assessed. Although significant group differences were found in several dexterity components, not all measures were pathological in all patients (above mean + 2SD threshold). For example, only 6 (of 10) patients showed pathological tracking error (Fig. 8a). Furthermore, only 3 patients (P03, P05, P06) showed pathological scores in all 6 measures. Thus, the presence of a pathological score in one variable did not always coincide with the presence of pathological scores in other measures. Neither did absence of one pathological score indicate absence in all other scores. The most common profile (in 4 patients) was a combination of five affected dexterity components: release duration, tracking error, number of overflow taps, omission rate and unwanted extra-finger-taps. These five components were increased compared to control thresholds.

### Relations and correlations with clinical measures

Individual dexterity profiles in patients (as described above) were not completely coherent with clinical scores. Among the five patients with a maximal ARAT score (P01, P02, P04, P08, P10), and therefore considered as having normal grip and gross-motor hand function, all were affected in at least one of the six measures. Four different profiles were observed: P04 had pathological scores in all six FFM measures. P10 had pathological scores in three measures: two in the multi-finger tapping task and one in the single finger tapping task (high number of overflow taps). P02 and P08 had pathological scores for two scores of the multi-finger tapping task, but not in the other tasks. Finally, P01 had pathological performance in the two measures of the force tracking task only.

**Fig. 9:**
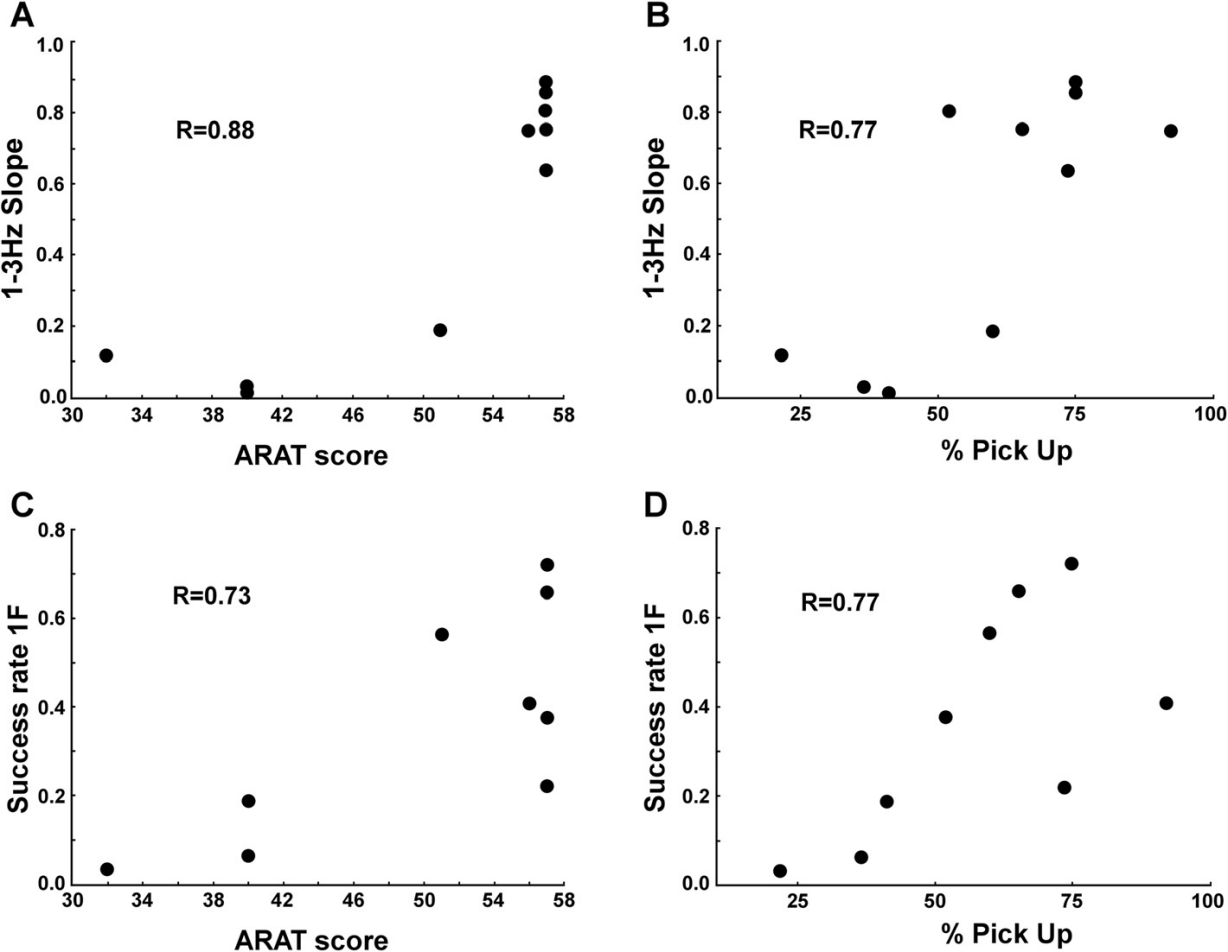
Correlations with clinical scores. **a**-**b** FFM single finger tapping (N = 10): **a** Correlation between 1-3Hz slope and the ARAT scores. **b** Correlation between 1-3Hz slope and the Moberg pick-up scores. **c**-**d** FFM multi-finger tapping (N = 9). **c** Correlation between success rate and the ARAT scores. **d** Correlation between success rate and the Moberg pick-up scores

We tested for correlations between the obtained performance measures in the FFM tasks and the ARAT or the Moberg pick-up test scores. Single finger tapping 1-3Hz slope appeared to be correlated with the ARAT score (Fig. 9a, R = 0.88; P = 0.0003) and with %Pick Up scores (Fig. 9b, R = 0.77; P = 0.004). The higher the slope during the single finger tapping task, the better were their ARAT or Pick Up scores. Multi-finger tapping success rate also appeared to be correlated with the ARAT score (Fig. 9c, R = 0.73; P = 0.03) and with %Pick Up (Fig. 9d, R = 0.77; P = 0.02). Again, a higher success rate in the multi-finger tapping task was found in patients with higher ARAT or %Pick Up scores. For the Finger force tracking task we did not find any correlations between performance variables and clinical measures. We also tested the inter-relations between the 6 measures used for the description of the dexterity profiles and we found four significant correlations among the 15 comparisons (Table 3). The strongest correlation was between 1-3Hz slope and the unwanted extra-finger-taps (1F) (R^2^ = 0.55).

**Table 3:** Pearson correlation coefficients (R^2^) between dexterity component scores

		Finger force tracking		Single finger tapping		Multi-finger tapping	
		Total error	RD	1-slope (1–3Hz)	OF 1 Hz	Omission rate	UEFT 1F
Finger force tracking	Total error						
	RD	0.38					
Single finger tapping	1-slope (1–3 Hz)	0.28	0.19				
	OF 1 Hz	0.10	0.11	0.27			
Multi-finger tapping	Omission rate	*0.49*	0.14	*0.47*	0.04		
	UEFT 1F	0.21	0.24	0.55	0.27	*0.47*	

Total error: finger force tracking error; RD: release duration; OF 1 Hz: number of overflow taps in 1Hz condition; UEFT 1F: number of unwanted extra-finger-taps during one-finger conditions. Italic correlation coefficients: significant at p < 0.05

## Discussion

We developed a novel device to quantify manual dexterity in a clinical context. This study shows that this device (the ‘FFM’) allows for the quantification of key control variables of manual dexterity in healthy subjects and in stroke patients. The patients tested in this study were able to use the FFM and performed most of the tasks suggesting adequate feasibility of the new method. Performance was impaired in all four visuo-motor tasks: patients showed less accurate force control, slowed finger tapping rate, more error in finger selection and in sequential finger tapping. We also found that patients were not equally affected across different components of manual dexterity which suggests the presence of individual dexterity profiles. These findings will be discussed in turn below.

### Feasibility

Healthy subjects had no problems performing the tasks and our mild-to-moderately affected hemiparetic patients were able to accomplish three out of the four visuo-motor tasks. However, the sequential finger tapping task proved difficult for stroke patients, presumably due to an inadequate (too high) task velocity. In terms of ergonomics, patients sometimes encountered problems in maintaining their fingers on the pistons, mostly for the little finger. This led some patients to look at their fingers, rather than at the screen, in order to replace them on the pistons. This problem could in part be due to decreased tactile sensitivity, shown by the Semmes-Weinstein test. The FFM allowed identification of decreased performance in at least one dexterity component in all patients (Fig. 8). Even in patients with maximal ARAT scores (N = 5) and in patients with normal Moberg Pick-up times (<18 s, N = 2) the FFM revealed deficient manual dexterity components, coherent with Lang et al. [42]. Although preliminary, given the small sample size, this suggests that the FFM may be more sensitive than other clinical measures in detecting underlying impairments important for dexterity in patients after stroke.

### Task performance: group differences between healthy subjects and hemiparetic patients

For the *tracking* task, which requires control of force, we found increased finger tracking error and longer release duration in patients, consistent with previous reports on power grip force control [18, 43]. Patients did not show higher force variability (CV of force) as previously reported [43]. However, this agrees with findings that did not show increased CV when stroke patients performed power grip force tracking at similar absolute forces as the controls [18].

The *sequential finger tapping* task, which requires motor learning of sequential digit selection, was too difficult for most patients. However, four patients were able to complete the task, but their performance was reduced compared to controls. While controls improved their success rate during the first sequence (sequence A) patients improved later in sequence C (Fig. 4b). This is consistent with studies showing intact but slowed motor learning capacity after stroke [27, 44].

The *single finger tapping* task, which requires explicit control of timing, revealed good temporal matching in patients for the 1 Hz and 2 Hz target frequencies, but a reduced tapping rate for the 3 Hz condition compared to controls. The performances measured were similar in all four fingers with no significant difference across fingers. Other studies have shown differences in maximal tapping rate between fingers [45], a measure we did not assess. Nonetheless, we assume that some patients had maximal tapping rate below 3 Hz since unable to follow this target rate. Other studies have also shown a decreased maximal finger tapping rate (and decreased regularity) in stroke patients [24, 26]. However, we did not find a decreased tapping regularity in patients: this could be due to differences in lesion localizations and tapping parameters used.

During *multi*-*finger tapping*, which requires on-line digit selection, patients were less accurate during one-finger or two-finger target taps (made more omissions and unwanted extra-finger- taps). The observed ‘neighborhood’ gradient of unwanted extra-finger-taps in control subjects is consistent with the known degree of independence of finger movements [46] and finger forces [47]. Unwanted extra-finger-taps were more frequent in patients and also followed the distance gradient. Again this shows decreased finger independence after stroke, consistent with previous reports [10, 22, 48]. Complementary to these previous observations based on purely kinematic measures, we show here that finger independence and its impairment in stroke also occurs in a task combining kinetic and kinematic constraints.

Together these findings show that the FFM allows quantification of different key parameters of manual dexterity with one and the same apparatus in a single one-hour session. The observed impairments of these key parameters in stroke patients with mild-to-moderate hemiparesis were partly consistent with previous reports, which confirms the relevance of these measures.

### Clinical correlations

Some of our measures correlated with clinical scales. Although these correlations need to be taken with caution (due to limited group size), they suggest that single finger tapping rate as well success rate in multi-finger tapping relate to hand functioning according to the ARAT, even though the ARAT showed a ceiling effect. These same two dexterity components also correlated with the Moberg pick-up score. This might point to common underlying control parameters, in particular timing (speed of execution) and digit selection (contrary to Raghavan et al. [22], who did not find any correlations between finger independence indices and clinical scores). The FFM thus provides some measures that correlate with clinical scales, which, however, needs to be confirmed in a larger sample size and with a larger variety of clinical scores.

### Individual dexterity profiles

Since the FFM allows for assessment of several different key control parameters it also provides the potential for obtaining individual profiles of impaired dexterity. The dexterity profiles varied in the patient group (Fig. 8) and patients were not equally affected in the various measures. For example, patient 09 had difficulty in releasing force, produced overflow and error taps, but showed similar accuracy in force tracking and tapping speed compared to controls. This patient therefore had difficulties in stopping and inhibiting movements in other fingers and would likely benefit from targeted training of these components.

The individual profiles (in Fig. 8) suggest that some of the measures are independent of each other, even if the omission rate and the capacity to increase the tapping rate moderately correlated to other measures (Table 3). This, however, will need further statistical elaboration in larger samples. Profiling of impairment should allow extraction of the most severely affected component(s) of dexterity and should permit individual optimization of rehabilitation protocols [49].

### Independence of finger movements and dexterity

In our view, independence of finger movements represents one functional aspect of dexterity. Four different FFM measures allow for characterization of the degree of finger independence. (i) The number of unwanted taps during single finger tapping, and during multi finger tapping, (ii) the success rate, (iii) the omission rate, and (iv) the distribution of unwanted extra-finger-movements. These four measures were impaired in our stroke patients, reflecting a reduced degree of finger individuation. However, single finger tapping is less complex than multi finger tapping: the latter requires various patterns of instantaneous effector selection. Indeed, the number of unwanted extra-finger-movements during multi-finger tapping was the most affected measure. This deficit in effector selection might be due to non-selective excitation and/or insufficient inhibition [9].

The distribution of unwanted extra-finger-taps (in single and two-finger taps) provided two additional insights into how independent finger movements are affected after stroke (Fig. 7). First, the ring finger was the least independent finger, replicating results from previous studies [9, 22]. Second, stroke patients had a similar ‘neighborhood’ gradient as control subjects, suggesting that stroke lesions do not affect this gradient and do not provoke finger-specific deficits (in this stroke group).

Independence of finger movements is not typically a clinical index. Previous studies on independence of finger movements in hemiparetic patients [22, 48], all based on kinematics measures, found small or no correlations with clinical hand function scales. Nevertheless, our measures of finger individuation correlated with the ARAT and the Moberg scores. This difference may relate to the fact that all our measures had a kinetic (force) component. Hence, finger individuation might usefully complement other functional scales, and its specific training may provide more efficient recovery than conventional rehabilitation [49].

### Limitations

The main limitation of our study concerns the group size: some findings (e.g., correlations between FFM measures and clinical scores) need to be confirmed with a larger sample that represents a broader range of lesion size and localization, as well as a more representative range of functional impairment. Nevertheless, even in this restricted sample we found clear-cut group differences and individually diverse dexterity profiles. Two methodological limits of the FFM were identified in the present study: the sequential tapping task was too difficult, due in part to lack of adjustable piston positioning, difficulty in maintaining the finger tips on the contact surface, and task velocity. These constraints may have affected certain performance measures. These issues will be addressed by simplifying the sequence task and by re-design of the FFM device.

## Conclusions

We developed a novel device, the FFM, to quantify key components of manual dexterity in a clinical setting. Use of the device, together with four visuo-motor tasks, was feasible in a group of hemiparetic stroke patients. On the group level, patients were significantly impaired in all four visuo-motor tasks compared to healthy control subjects. Patients showed less accurate finger force control, slowed finger tapping rate, more error in finger selection and in sequential finger tapping. Moreover, the four tasks allowed for individual profiling of post-stroke impairment in dexterity. This suggests that this new device provides a more complete and more sensitive assessment of manual dexterity than previous devices or clinical scores.
